# Differential Diagnosis of Cysts and Granulomas Supported by Texture Analysis of Intraoral Radiographs

**DOI:** 10.3390/s21227481

**Published:** 2021-11-10

**Authors:** Elżbieta Pociask, Karolina Nurzynska, Rafał Obuchowicz, Paulina Bałon, Daniel Uryga, Michał Strzelecki, Andrzej Izworski, Adam Piórkowski

**Affiliations:** 1 Department of Biocybernetics and Biomedical Engineering, AGH University of Science and Technology, 30-059 Krakow, Poland; paulina.x.balon@gmail.com (P.B.); izwa@agh.edu.pl (A.I.); 2 Department of Algorithmics and Software, Silesian University of Technology, 44-100 Gliwice, Poland; Karolina.Nurzynska@polsl.pl; 3 Department of Diagnostic Imaging, Medical College, Jagiellonian University, 31-501 Krakow, Poland; rafalobuchowicz@su.krakow.pl; 4 Department of Oral Surgery, Medical College, Jagiellonian University, 31-155 Krakow, Poland; duryga@op.pl; 5 Institute of Electronics, Lodz University of Technology, 93-590 Lodz, Poland; michal.strzelecki@p.lodz.pl

**Keywords:** texture features, classification, periapical lesions, intraoral radiography, tSNE, granulomas, cysts

## Abstract

The aim of this study was to evaluate whether textural analysis could differentiate between the two common types of lytic lesions imaged with use of radiography. Sixty-two patients were enrolled in the study with intraoral radiograph images and a histological reference study. Full textural analysis was performed using MaZda software. For over 10,000 features, logistic regression models were applied. Fragments containing lesion edges were characterized by significant correlation of structural information. Although the input images were stored using lossy compression and their scale was not preserved, the obtained results confirmed the possibility of distinguishing between cysts and granulomas with use of textural analysis of intraoral radiographs. It was shown that the important information distinguishing the aforementioned types of lesions is located at the edges and not within the lesion.

## 1. Introduction

Apical periodontitis and root canal treatment are two common causative factors of reduced periapical bone density [1]. Localized inflammatory reaction with subsequent bone loss is secondary to the action of blood-derived macrophagic cells, which represents a response to stimuli such as mechanical irritation or bacterial inoculation. This form of bone change is represented in intraoral radiography (IR) by areas of radiolucency [2,3]. In the case when such a change is adhered to the tooth, its presence bespeaks an inflammatory process or the formation of granulation tissue. Those changes are different from the clinical point of view [4]. Histological assessment has proven that periapical granulomas on IR are relatively small, with poorly defined borders; moreover, they are more prevalent than larger, often well-defined cystic lesions [5,6]. The presence of lesions is associated with pain and clinical picture of tooth morbidity. IR is the main technique to support the processes of diagnosis and clinical decision making [7,8]. However, geometrical distortions limit detection of periapical changes overlying anatomical structures known as “anatomical noise”: differences in bone density and lesion shape [9,10,11]. Where detected, periapical radiolucent changes caused by granulomas and cysts look very similar when assessed in IR studies [12]. Therefore, differentiation between cystic lesions and periapical granulomas is still a great dilemma in dentistry, as the sensitivity and specificity of diagnostic processes supported by IR are not satisfactory [13]. In dentistry overall, the healing prognoses for granulomas and cysts, and therefore further treatment, are different [14]. In endodontics, radiolucent periapical areas were discovered to be a prognostic factor for the success rate of the canal treatment. Therefore, accurate diagnosis is highly welcomed by the clinical community, although this contemporarily still relies on histologic examination, with poor outcomes for intraoral radiography and unsatisfactory results for cone beam tomography (CBCT) [15,16]. However, CBCT scans find their application in determining the key parameters of temporomandibular joint fissures, which allow verifying whether the ponds look and work properly [17].

Intraoral radiological images can be transformed into digital data and then processed by image analysis methods. Although granulomas and cysts look similar in IR images, their object textures should reflect the differences in tissue degradation. Therefore, we propose building a classification model that takes the feature calculated from texture operators as the input. This approach is novel for described purposes, but we have already proved its usability. For instance, bone loss caused by periodontitis [18], bone healing [19,20], and caries [21] were estimated by the use of texture feature maps for retrospective analysis of radiographs. Texture entropy proved to be a good feature for investigating the bone healing process [22] in a similar way as fractal dimensions describing the bone healing region of interest [23]. Since previous methods of distinguishing cysts from granulomas were not satisfactory, we decided to investigate textural features as a method of differentiating the above lesions, given the success of texture analysis in a similar field. Thus, the contribution of this paper was the development of a new method that allowed automatic analysis of intraoral radiograms with the use of selected texture features that enable discrimination between cysts and granulomas via reflecting their anatomical structure.

The work is organized as follows. Section 2 describes the IR image database and gives a precise overview of texture operators considered for differentiation between granulomas and cysts. Next, we present the experiments concerning the selection of the texture operators for classification of those changes in Section 3. Then, discussion of the achieved outcomes is given in Section 4.

## 2. Materials and Methods

A set of periapical histological specimens extracted during tooth extraction periapical management connected with tissue debridement was referred for histological evaluation. Tissue specimens embedded in solution of formaldehyde were transferred to the Department of Pathophysiology (CM-UJ). Furthermore, a database of IR images depicting the aforementioned changes was prepared. Additionally, a manual annotation showing the section of changed tissue was prepared to complement each image. This information allowed splitting the selected region into smaller patches. Next, texture operators computed features describing characteristics of the patches supplied as an input for the classification. The details of the applied image analysis techniques and proposed classifiers are presented in this section.

### 2.1. Ethics Approval and Consent to Participate

The study protocol was designed in accordance with the guidelines of the Declaration of Helsinki and the Good Clinical Practice Declaration Statement. Particular care was taken to ensure the safety of personal data, and all images were anonymized before processing. Written consent for the publication of clinical data and anonymous clinical images was obtained from the local Scientific Committee of the Jagiellonian University (no 102.6120.25.2017, dated 21 December 2017). Written informed consent of the Scientific Board of the Department of Dentistry was obtained for patient data processing.

### 2.2. Image Database

**Figure 1:**
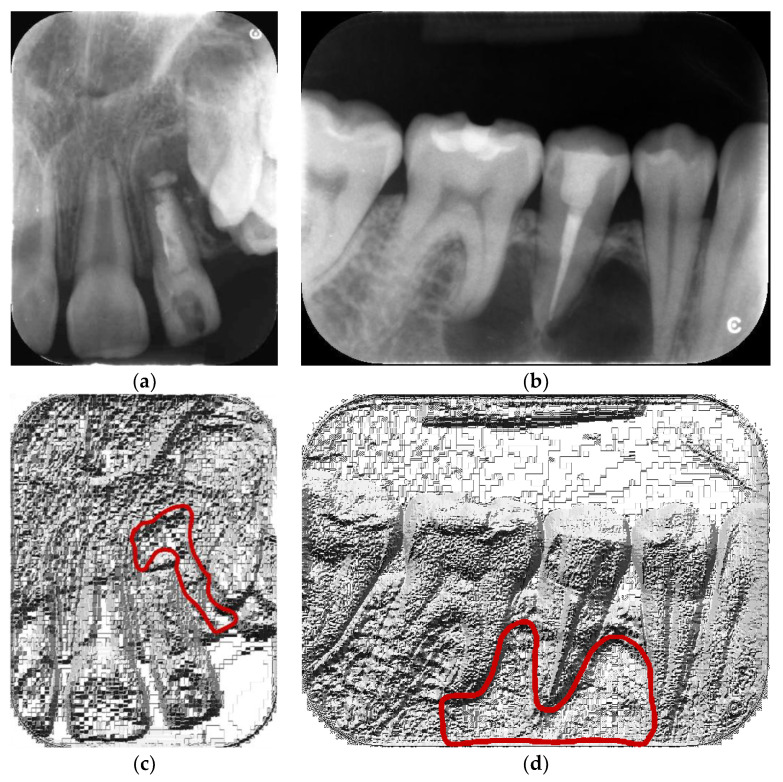
Example of lossy compression artifacts on local binary pattern (LBP) feature maps (**c**,**d**) calculated for analyzed JPG images (**a**,**b**). Lesions are depicted with red lines.

The image database consisted of anonymized, digitalized IR Images from patients who attended the dental clinic presenting lytic lesions from 2015 to 2018. The images were selected from the institutional picture archiving and communication system (PACS), which used lossy JPG without scale preservation as the storage format (Figure 1).

**Figure 2:**
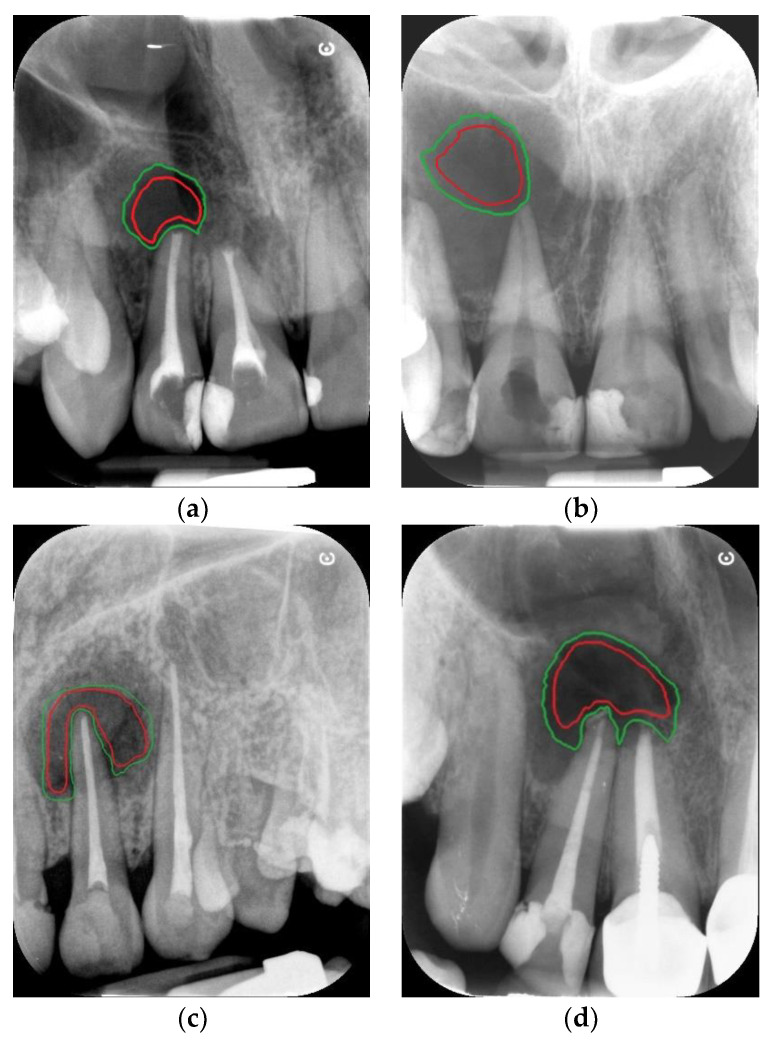
Example of ROI outlines: for granulomas (**a**,**c**), for cysts (**b**,**d**). Legend: red for the lesion interior outline, green for the outline including the lesion edges.

The documentation covered images of 62 patients of both sexes aged 34–61 years with histological evaluation in the diagnostic process. Periapical radiographs were obtained using a dental X-ray system (Gendex Kavo 765 DC Intraoral X-Ray System, Biberach, Deutschland). Data were acquired at 65 kV and 7 mA with a mean exposure time of 0.1 s and recorded on phosphor plates with a secondary readout of five detectors (CS 7600, Carestream Dental LLC, Atlanta, GA, USA) connected to a Kamsoft computer system. The resolution of the image varied from min. 490 × 649 to max. 1528 × 2024 pixels depending on the recorded details. There were 23 samples presenting granulomas and 39 presenting cysts. For each lytic lesion, two ROIs were manually annotated on each image, the first containing the maximum area of the lesion interior and the second containing an extension of this area to include the edge of the lesion (Figure 2).

### 2.3. Image Processing Techniques

In the presented work, more than 10,000 texture features were computed from the determined ROIs, and finally, 6836 features were accepted for further analysis for each lesion, cysts and granulomas. The analyses were performed using dedicated qMaZda software [24,25].

We selected texture operators to extract qualitative information from the images. Texture operators represent a kind of complex function that replaces a two-dimensional manifold by a set of parameters that fully describe its content. Most such operators derive information addressing one aspect of texture quality (e.g., contrast). The others use histograms describing a distribution of edges and their location in the image. Below, the chosen methods are described in detail, since we would like to make this article self-sufficient.

#### 2.3.1. First Order Features

First order features constitute a method that derives qualitative measures from the image intensity distribution. To be precise, having an image, *I*, that represents a discrete, two-dimensional function of two variables, x and y, it is possible to calculate a histogram representing the probability of occurrence of each intensity value as:

(1) $$H\left(i\right)=\frac{1}{M\times N}\overset{N-1}{\underset{y=0}{\sum }}\overset{M-1}{\underset{x=0}{\sum }}\{\begin{array}{c} 1\;\;\;\;\;\;I\left(x,y\right)=i\\ 0\;\;\;\;\;otherwise, \end{array}$$

where *N* and *M* are the image height and width, respectively, and *G* defines the number of intensity levels, *i*—when a greyscale image is considered, *G* is in the range 0–255.

It is possible to calculate six parameters for a complete image description knowing the histogram. The value of average intensity *µ* tells whether the image is dark or bright. The variation $\sigma^{2}$ in shades reflects the information of image uniformity. Next, the skewness corresponds to the lack of symmetry in the histogram, and kurtosis takes 0 for a normal distribution. Energy is a possible measure of contrast, and entropy measures the lack of predictability. Equations (2)–(7) give the formulas for those parameters in the order they were mentioned here.

(2) $$\mu =\overset{G}{\underset{i=0}{\sum }}i\times H\left(i\right)$$

(3) $$\sigma^{2}=\overset{G}{\underset{i=0}{\sum }}{\left(i-\mu \right)}^{2}\times H\left(i\right)$$

(4) $$FOF_{skewness\;}=\sigma^{-3}\overset{G}{\underset{i=0}{\sum }}{\left(i-\mu \right)}^{3}\times H\left(i\right)\;$$

(5) $$FOF_{kurtosis\;}=\sigma^{-4}\overset{G}{\underset{i=0}{\sum }}{\left(i-\mu \right)}^{4}\times H\left(i\right)-3$$

(6) $$FOF_{energy\;}=\overset{G}{\underset{i=0}{\sum }}H{\left(i\right)}^{2}$$

(7) $$FOF_{entropy\;}=-\overset{G}{\underset{i=0}{\sum }}H\left(i\right)log_{2}H\left(i\right)$$

#### 2.3.2. Second Order Features

Second order features regard the spatial relations between the intensity levels in the image [26]. The cooccurrence matrix *p* stores the correlation between the pixel intensities. It is a square matrix of resolution *G* × *G* in which each entry tells a probability of occurrence of intensity levels that indexes the entry. It is possible to decide the distance between neighboring pixels. When one needs the method to be rotation invariant, not only the horizontal but the vertical and diagonal neighborhoods are used for matrix calculation. There are 14 Haralick features calculated from this matrix, which describe among other features the contrast, homogeneity and correlation of the image. Equations (8)–(11), respectively, describe these features, which are the most widely used parameters; however, in our experiments, all 14 were considered.

(8) $$COM_{contrast}=\overset{G}{\underset{i=0}{\sum }}\overset{G}{\underset{j=0}{\sum }}{\left(i-j\right)}^{2}p\left(i,j\right)$$

(9) $$COM_{correlation}=\overset{G}{\underset{i=0}{\sum }}\overset{G}{\underset{j=0}{\sum }}\frac{i\times j\times p\left(i,j\right)-\mu_{x}\mu_{y}}{\delta_{x}\delta_{y}}$$

(10) $$COM_{homogenity}=\overset{G}{\underset{i=0}{\sum }}\overset{G}{\underset{j=0}{\sum }}\frac{p\left(i,j\right)}{1+{\left(i-j\right)}^{2}}$$

(11) $$COM_{entropy}=\overset{G}{\underset{i=0}{\sum }}\overset{G}{\underset{j=0}{\sum }}p\left(i,j\right)log_{2}\left(p\left(i,j\right)\right)$$

#### 2.3.3. Run Length Matrix

Another method analyzes the lengths of pixels with similar illuminance coappearing next to each other in one line and calls it runs [27]. It constructs the run-length matrix *r*, which counts the number of runs of each illuminance value for chosen lengths in the range 1–*L*. To achieve rotation invariance, the method considers several angles for run analysis. The foundation of this method is the finding that short runs characterize textures of good quality, while long runs correspond to coarse texture.

The method introduces five parameters. One parameter emphasizes short runs and obtains high values for textures of high quality. Another returns high values for coarser textures, as it concentrates on long runs. A third represents the possibility of finding nonuniformity of grey-level distribution and run lengths. Finally, the run percentage gives information about the overall image quality. Equations (12)–(16), respectively, show how to compute those parameters from the run-length matrix, with *n_r_* standing for the number of runs. It is possible to calculate up to eleven parameters when considering the findings presented in [28], as we did in this work.

(12) $$RLM_{shortRunEmphasis}=\frac{1}{n_{r}}\overset{G}{\underset{i=0}{\sum }}\overset{L}{\underset{j=1}{\sum }}\frac{r\left(i,j\right)}{j^{2}}$$

(13) $$RLM_{longRunEmphasis}=\frac{1}{n_{r}}\overset{G}{\underset{i=0}{\sum }}\overset{L}{\underset{j=1}{\sum }}r\left(i,j\right)\times j^{2}$$

(14) $$RLM_{grayLevelNonUniformity}=\frac{1}{n_{r}}\overset{G}{\underset{i=0}{\sum }}(\overset{L}{\underset{j=0}{\sum }}r\left(i,j\right))$$

(15) $$RLM_{runLengthNonUniformity}=\frac{1}{n_{r}}\overset{L}{\underset{j=1}{\sum }}(\overset{G}{\underset{i=0}{\sum }}r\left(i,j\right))$$

(16) $$RLM_{runPercentage}=\frac{n_{r}}{M\times N}$$

#### 2.3.4. Grey-Tone Difference Matrix

Another approach for texture description presents the grey-tone difference matrix [29], which is created using knowledge of how the human visual system perceives texture. Here, similarly as in other techniques, an additional structure is present. The matrix stores information about the absolute illumination changes between a central pixel and the average illumination of its neighborhood $I_{W}\;$ in a square window with sides of length *W*. Each entry of the matrix describes a sum of differences for a chosen intensity level, as presented in Equation (17):

(17) $$s\left(i\right)=\overset{M-1}{\underset{x=0}{\sum }}\overset{N-1}{\underset{y=0}{\sum }}\left|I\left(x,y\right)-I_{w}\right|$$

There are defined five texture parameters. One describes the texture coarseness and is related to the average texture grain size. Another gives information about the contrast in the image. The texture business parameter describes spatial frequencies. The fourth parameter gives details on image complexity and whether the image contains many edges or not. Finally, the last parameter gives data about how well the primitives are visible in the image by the strength feature. Equations (18)–(22), respectively, give the formulas for the calculation of these parameters.

(18) $$GTDM_{coarseness}={\left(\in +\overset{G}{\underset{i=0}{\sum }}H\left(i\right)s\left(i\right)\right)}^{-1}$$

(19) $$GTDM_{contrast}=\left[\frac{1}{G\times \left(G+1\right)}\overset{G}{\underset{i=0}{\sum }}\overset{G}{\underset{j=0}{\sum }}H\left(i\right)H\left(j\right){\left(i-j\right)}^{2}\right]\left[\frac{1}{n}\overset{G}{\underset{k=0}{\sum }}s\left(k\right)\right]$$

(20) $$GTDM_{business}=\frac{\sum_{i=0}^{G}H\left(i\right)\times s\left(i\right)}{\sum_{i=0}^{G}\sum_{j=0}^{G}\left|i\times H\left(i\right)-j\times H\left(j\right)\right|}\;\;H\left(i\right),H\left(j\right)\neq 0$$

(21) $$GTDM_{complexity}=\overset{G}{\underset{i=0}{\sum }}\overset{G}{\underset{j=0}{\sum }}\frac{\left|i-j\right|}{n\times \left(H\left(i\right)+H\left(j\right)\right)}\left[H\left(i\right)\times s\left(i\right)+H\left(j\right)\times s\left(j\right)\right]\;\;H\left(i\right),H\left(j\right)\neq 0\;$$

(22) $$GTDM_{strength}=\frac{\sum_{i=0}^{G}\sum_{j=0}^{G}(\left(H\left(i\right)+H\left(j\right)\right){\left(i-j\right)}^{2}}{\in +\sum_{i=0}^{G}s\left(i\right)}\;\;H\left(i\right),H\left(j\right)\neq 0$$

∈ stands for a very small value, which prevents from division by zero. It is also necessary to know the number of pixels used for matrix calculation: *n* = (M − 2W) (N − 2W).

#### 2.3.5. Local Binary Patterns

Texture is a two-dimensional phenomenon in which illuminance spatial relations and contrast play a crucial role. The quality of the latter two features describes a joint distribution of intensity levels on a circularly symmetric neighbor set. Unlike other methods, here, for each pixel, a binary code is calculated, and a histogram of those codes is a description of the image’s content [30].

The pixel neighborhood is sampled *p* times on a circumference with radius *R*. The number of sampled points depends on the needs; in many presented scenarios, it has taken eight. Each sampled illuminance $g_{p}$ value is compared with the central one $g_{c}=I\left(x,y\right)$ and coded as 0 for a smaller intensity and 1 otherwise, according to the formula:

(23) $$lbp\left(z\right)=\{\begin{array}{c} 1,\;\;\;\;z\geq 0,\\ 0,\;\;\;\;z<0. \end{array}$$

Next, a weighted sum of all sampled points in the neighborhood gives the local binary pattern code:

(24) $$LBP_{P,R\left(x_{c},y_{c}\right)}=\overset{P-1}{\underset{p=0}{\sum }}lbp\left(g_{p}-g_{c}\right)\times 2^{P}$$

### 2.4. Statistics 

The texture features listed in Section 2.2 were generated for monochrome images with gray levels ranging from 0 to 255.

Obtaining images from different cameras or using different settings can generate the occurrence of undesirable differences in brightness and contrast, and the use of appropriate normalization can overcome this problem. In this study, parameter groups were calculated for four cases: D—without normalization, raw data; S—with normalization <μ−3σ, μ+3σ>; M—minimum and maximum values of grey levels in ROI defined a new range; *n*-percentiles of gray levels in the histogram were calculated, and the new range was defined by the 1st and 99th percentile <p1, p99>. The impact of normalization was presented in [31].

The Shapiro–Wilk test was used to assess normal distribution of continuous variables. We verified whether multiple samples from populations had equal variances using Bartlett’s test. Continuous data were presented as mean ± standard deviation or as medians (interquartile range, IQR) and were compared using Student’s t-test or the Mann–Whitney U test, as appropriate (see Table 1). Categorical data were presented as numbers (percentages), and Fisher’s exact test was used to compare categorical results.

The following statistically based feature selection methods were used to select the best subset of input variables (texture features). It was desirable to reduce the number of input variables (6836 features) to find the best potential predictors, thus improving the performance of the model. To perform the best selection, the values of Spearman’s rho correlation coefficients (rho Spearman ≥ 0.4) and the Mann–Whitney U test (*p* values < 0.05) were used. Regarding Spearman’s rho, the strongest correlation found was taken as the cutoff value. In our study, it was around 0.4, which allowed us to conclude that this value may have been higher after data standardization. 

Logistic regression was used to build a model for discrimination between cysts and granulomas based on the studied texture features. In our case, we considered multiple predictor variables (texture features), and the logistic function was:

(25) $$\log\left[\frac{p}{1-p}\right]=b0+b1\times x1+b2\times x2+\cdots +bn\times xn$$

where *b*0 and *b*1 are the regression beta coefficients. A positive *b*1 indicated that increasing *x* was associated with increasing *p.* Conversely, a negative *b1* indicated that increasing *x* was associated with decreasing *p*. The quantity log(*p*/(1 − *p*)) is the logarithm of the odds and reflects the likelihood that the event occurs. Technically, odds are the probability of an event divided by the probability that the event will not take place [32]. In our case, *p* was the probability of granulomas occurring given *xn* chosen texture features (see Table 2).

Additionally, the t-SNE model as a nonlinear and unsupervised technique [33] was used for multivariate data mining and visualization.

## 3. Results

In order to reduce the number of parameters, the correlation of features was checked. It was observed that the ROI for the first dataset, with the interior of the lesion, was significantly higher than that for the second dataset, which was extended by the edges. The correlation coefficient between texture features was mostly in the range 0.9–1.0. This made it necessary to discard the first one (ROI marked in red in Figure 2), as it was not possible to select independent features. Because of this observation, only the parameters calculated for the ROI containing the pathological lesion contours were analyzed in the remainder of this study. The potential input variable for a target variable was identified and collected as shown in Table 1.

Based on the Spearman correlation and *p*-value (see Table 1), the most suitable input variable were chosen to perform a logistic regression. We created four models to predict the class of cysts or granulomas given multiple predictor variables (see Table 2). Table 2 shows the beta coefficient estimates associated to each predictor variable and their significance levels. The smaller the *p*-value, the more significant the estimate was. To measure the association between a predictor variable and the outcome variable, the odds ratio (OR) with 95%CI was calculated for each predictor variable. Akaike’s information criteria (AIC) and McFadden’s R2 were calculated to assess the quality of the built models. McFadden’s R2 is defined as:

(26) $$1-\left[\frac{\ln\left(LM\right)}{\ln\left(L0\right)}\right]\;\;\;\;$$

where ln(*LM*) is the log likelihood value for the fitted model and ln(*L*0) is the log likelihood for the null model with only an intercept as a predictor. Next *p*-value for R2 was calculated using a chi-square distribution.

The main idea of AIC is to penalize the inclusion of additional variables in a model. It adds a penalty that increases the error when including additional terms. The lower the AIC, the better the model.

Model I seemed to be the best fitted, even though the parameters included in this model had Spearman coefficients below 0.4. Furthermore, the best predictor variable could have been the texture feature YS6GlcmZ4Entropy. The odds that a lesion was a granuloma were 57% higher (OR = 1.57, 95% CI = 1.115–2.483, *p* = 0.03) if this feature increased by 1 unit. 

**Figure 3:**
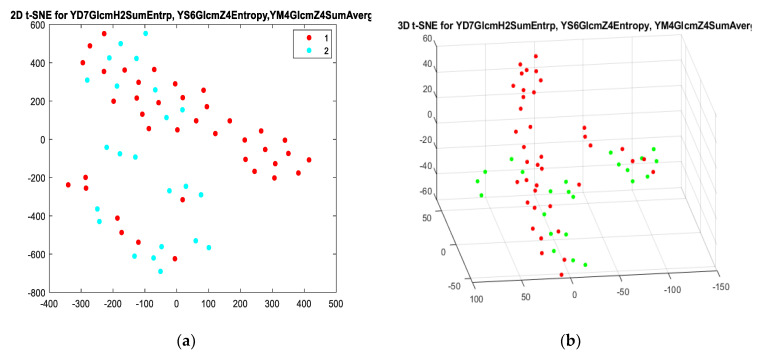
Two-dimensional t-SNE (**a**) and 3D t-SNE (**b**) for model I for perplexity = 20. In (**a**), class 1 (red dots) represents cysts, and class 2 (blue dots) represents granulomas. In (**b**) red dots represent cysts, and green dots represent granulomas. The aggregation of red dots suggested that the chosen texture features could be used as a predictor to differentiate these two lesions.

**Figure 4:**
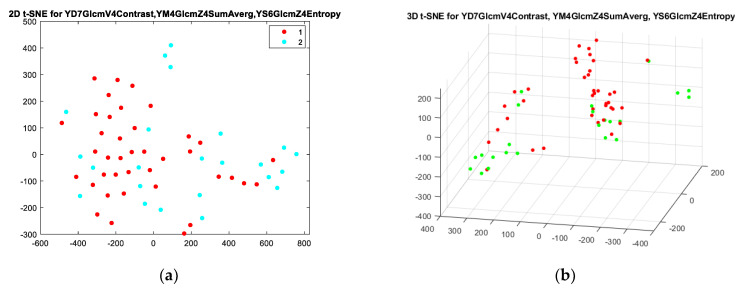
Two-dimensional t-SNE (**a**) and 3D t-SNE (**b**) for model IV for perplexity = 20. In (**a**), class 1 (red dots) represents cysts, and class 2 (blue dots) represents granulomas. In (**b**), red dots represent cysts, and green dots represent granulomas. In both figures, the red dots tended to accumulate in a particular area of space.

Additionally, 2D and 3D t-SNE were used for all four models, but only model I and model IV (Figure 3 and Figure 4) showed the highest tendency in differentiating periapical lesions. In all generated t-SNE models, class 1 represented texture parameters describing cysts, and class 2 represented granulomas. Euclidean distance, the “exact” algorithm, and Perplexity 10, 15, 20, and 25 were used as parameters to implement t-SNE models. The Kullback–Leibler divergence between the distributions, which modelled the data X and the embedding Y for different values of the perplexity parameter, was also checked, and the smallest results were obtained for perplexity = 20.

## 4. Discussion

The idea of conducting IR radiogram postprocessing in order to find radiographic features of granulomas and cystic lesions is not new. Possible differentiation of histologically verified cysts and granulomas for a relatively large group of patients was introduced in [34], in which the use of analysis of minimal and maximal radiographic densities obtained from digital radiographs was proposed. The work concluded that to some extent the differentiation between periapical cysts and granulomas was possible, but a definite distinction was not feasible. Digital analysis of radiological images is an objective tool of examination in comparison with visual evaluation of radiograms performed in clinical conditions. A similar idea with a different approach was introduced by White [35]. As in our study, the authors used digitized images and evaluated a technique based on the analysis of histograms for detection of the granulomas and cysts.

Although classification was only possible when the imagery was supported with additional information concerning the clinical state of the patient, Shrout used the region of interest centered at the periapical lesion and verified its content by histogram shape analysis of radiograms [6]. The classic image processing methods (top hat, erosion, and opening) were applied to intraoral radiographs for analysis of periapical lesion healing [36].

To our knowledge, this cohort study was the first to include texture feature analysis for automatic discrimination between cysts and granulomas based on intraoral images, although texture feature map correlation has been used for cyst and granuloma detection in magnetic resonance [37] and computed tomography [38].

The study analyzed 6836 texture parameters for two groups, one containing ROIs of lesions with edges and the other containing only the interiors of lesions, which were subjected to a reduction process to obtain the best subset of predictor variables.

During the parameter reduction step, it was observed that within the texture features obtained for ROIs containing only the interior of the lesion, there was a very strong cross-correlation of parameters, and it was not possible to select independent features. In the case of the set containing results for ROIs of the lesion including the edges, this problem did not occur, indicating that the relevant information must be contained in the lesion contour. The lesion outline is the margin between healthy tissue and the lesion. It is different in the cases of cysts and granulomas; granulomas creates a fibrous capsule, while radicular cysts are lined with epithelium [1]. This feature influences textural analysis of the given lesion. In addition, high cross-correlation was also observed within the group of texture features obtained from the run length matrix, indicating that the structure was isotropic. Finally, 11 parameters were typed based on the values of Spearman’s rho correlation coefficients and the Mann–Whitney U test.

Logistic regression, 2D and 3D t-SNE models were created for the reduced groups. The best results were obtained for model I, created for data with significant values from the Mann–Whitney U test and correlation coefficient closes to 0.4. Considering model I, it can be noted that one parameter, YS6GlcmZ4Entropy, showed especially strong correlation with the outcome variable. The odds that a given lesion was a granuloma were 57% higher (OR = 1.57, 95% CI = 1.115–2.483, *p* = 0.03) if this parameter increased by one unit. This may be due to the fact that granuloma lesions are more differentiated at the margins than cysts, which end in an organized structure.

## 5. Conclusions

It was demonstrated that the proposed method enabled discrimination between cysts and granulomas in intraoral radiograms by implementing texture analysis. The results obtained at this stage were satisfactory and indicated the possibility of differentiating cysts and granulomas. This study identified texture features that may be key in differentiating between granulomas and cysts at lesion margins. The high correlation of the obtained results with reference materials indicated the possibility of exact differentiation by anatomical features of given lesions, although images distorted by lossy compression were analyzed.

The key evidence for the correlation of the observed data was that no correlation existed for ROIs involving only internal change, while it existed for ROIs with boundaries. The most important information about the differentiation of lesions was found in the border of the lesion. This conclusion provides a basis for further research, which will be conducted on a larger scale, based on the results obtained herein, using noncompressed and rescaled image data.

## Figures and Tables

**Table 1:** The potential input variables for a target variable identified using statistically based methods.

Variable	Cysts Mean (sd)	Granulomas Mean (sd)	Cysts Median (IQR)	Granulomas Median (IQR)	*p*-Value	Spearman’s Rho
YD7GlcmH2SumEntrp	58719.60 (366693.20)	478553.48 (940850.77)	1.73 (0.32)	1.81 (13349.24)	0.0150	0.3125
YD7GlcmV4Contrast	49449.81 (210805.80)	430143.48 (870210.86)	3.95 (2.56)	8.07 (81594.76)	0.0006	0.4375
YD6GlcmN3Entropy	17001.70 (106164.69)	426523.28 (961445.21)	1.78 (0.48)	1.89 (0.38)	0.0372	0.2677
YS7HistDomn01	52.74 (8.39)	59.39 (11.25)	51.00 (11)	57.00 (14.5)	0.0155	0.3109
YS6GradKurtosis	147.13 (119.33)	253.26 (201.87)	121.20 (155.53)	262.02 (215.68)	0.0475	0.2546
YS6GlcmZ4Entropy	2.57 (0.19)	311306.55 (739638.40)	2.59 (0.26)	2.75 (0.32)	0.0039	0.3704
YM4ArmTeta3	0.39 (0.06)	0.35 (0.06)	0.39 (0.08)	0.34 (0.06)	0.0091	−0.3349
YM4GlcmZ4SumAverg	204855.85 (568030.58)	741182.62 (1130874.17)	13.31 (3.9)	15.51 (1527485.65)	0.0011	0.4189
YN6GlcmH1AngScMom	0.01 (<0.001)	0.01 (<0.001)	0.01 (0.006)	0.01 (0.002)	0.0091	−0.3349
YLbpOc4n6	0.08 (0.02)	0.09 (0.02)	0.08 (0.02)	0.10 (0.03)	0.0073	0.3442
MorMzNi	58.74 (32.51)	37.35 (15.74)	49.00 (44)	37.00 (24)	0.0114	−0.3248

**Table 2:** Comparison of built logistic regression models.

Model	Predictor Variables (Texture)	Estimate	*p*-Value	OR	CI (95%)	R2 (McFadden)	*p*-Value for R2	AIC
I	YD7GlcmH2SumEntrp	1.15 × 10^−6^	0.033	1	(1.00, 1.00)	0.30	2.03 × 10^−5^ 2.03 × 10^−5^	65.34
	YS6GlcmZ4Entropy	4.51	0.0309 0.031	1.57	(1.115, 2.483)			
	YM4GlcmZ4SumAverg	8.7 × 10^−7^	0.048	1	(1.000, 1.000)			
II	YM4GlcmZ4SumAverg	9.10 × 10^−7^	0.024	1	(1.00, 1.00)	0.27	5.17 × 10^−5^	67.29
	YLbpOc4n6	40.5	0.018	57.445	(2.585, 2467.913)			
	MorMzNi	−4.6 ×10^−2^	0.013	0.995	(0.991, 0.998)			
III	YS6GradKurtosis	5.43 × 10^−3^	0.009	1.001	(1.000, 1.001)	0.26	2.45 × 10^−5^	66.54
	YS6GlcmZ4Entropy	3.93	0.048	1.482	(1.093, 2.273)			
IV	YD7GlcmV4Contrast	1.21 × 10^−6^	0.074	1	(1.000, 1.000)	0.29	3.39 × 10^−5^	66.41
	YM4GlcmZ4SumAverg	8.24 × 10^−7^	0.038	1	(1.000, 1.000))			
	YS6GlcmZ4Entropy	3.46	0.077	1.413	(1.047, 2.155)			

## Data Availability

Data can be made available on request by contacting the corresponding author.
